# Drivers’ Visual Attention Characteristics under Different Cognitive Workloads: An On-Road Driving Behavior Study

**DOI:** 10.3390/ijerph17155366

**Published:** 2020-07-25

**Authors:** Yanli Ma, Shouming Qi, Yaping Zhang, Guan Lian, Weixin Lu, Ching-Yao Chan

**Affiliations:** 1 School of Transportation Science and Engineering, Harbin Institute of Technology, Harbin 150090, China; mayanli@hit.edu.cn (Y.M.); shouming1991@163.com (S.Q.); 2 California PATH, University of California, Berkeley, CA 94804, USA; cychan@berkeley.edu; 3 School of Architecture and Transportation Engineering, Guilin University of Electronic Technology, Guilin 541004, China; lianguan@guet.edu.cn; 4 School of Electronic Engineering and Computer Science, Peking University, Beijing 100871, China; lweixin@pku.edu.cn

**Keywords:** on-road experiment, cognitive workload, visual fixation characteristics, visual transition characteristics, entropy method, Markov processes

## Abstract

In this study, an on-road driving experiment was designed to investigate the visual attention fixation and transition characteristics of drivers when they are under different cognitive workloads. First, visual attention was macroscopically analyzed through the entropy method. Second, the Markov glance one- and two-step transition probability matrices were constructed, which can study the visual transition characteristics under different conditions from a microscopic perspective. Results indicate that the fixation entropy value of male drivers is 23.08% higher than that of female drivers. Under the normal driving state, drivers’ fixation on in-vehicle systems is not continuous and usually shifts to the front and left areas quickly after such fixation. When under cognitive workload, drivers’ vision transition is concentrated only in the front and right areas. In mild cognitive workload, drivers’ sight trajectory is mainly focused on the distant front area. As the workload level increases, the transition trajectory shifts to the junction near the front and far sides. The current study finds that the difference between an on-road test and a driving simulation is that during the on-road driving process, drivers are twice as attentive to the front area than to the driving simulator. The research provides practical guidance for the improvement of traffic safety.

## 1. Introduction

With the improvement of vehicle automation technology, we can see human-machine co-driving or partially automated systems existing in the foreseeable future. The world’s major automobile manufacturers will continue the development of driver assistance systems, which would improve the safety and comfort of drivers and moreover, provide better protection as well. However, with the assistance of vehicle automation, drivers can feel freer to get distracted or bold to disengage from driving. Obviously, these automated systems interfere with drivers to make safe decisions during important moments and can result in misjudged safety risks [1]. As in other fields, driving safety increasingly depends on the comprehensive performance of human-machine interaction and automation technology and requires in-depth knowledge of the driver. Designers should not assume that automation can seamlessly replace human drivers or that drivers can safely adapt to the limitations of automation [2]. A driver’s concentration level directly affects their driving safety. Cognitive attention is mainly responsible for processing the external information received by human-being’s brains, transforming it into inner activity and then dominating human driving behaviors. The interference with the cognition capacity affects everything from task planning strategy to driver operation control. The current state-of-the-art driving assistance systems are not equipped to fully comprehend these issues and certain needs must be further explored to develop such knowledge. Finding ways to reduce or accommodate for driver distraction is a critically important issue in road safety. This study investigates the visual attention fixation and transition characteristics of drivers under different cognitive workloads to further understand their behavior.

Driving demand has been described as a satisfactory task [3,4], which means that the goal is to drive fully enough to satisfy the driver and where possible, balance between conflicting goals, such as task difficulty, perceived risk, and safety. Increasing vehicle automation may eliminate many driving needs, exacerbating these trends, which may induce drivers to engage in distracting activities. Distraction occurs when drivers transfer their attention from the driving task to focus on other activities. This has become an important issue on the road safety agenda in many countries around the world. Recent evidence from the United States shows that driving distraction and inattention are more important than other factors that are generally considered to be critical to the cause of a crash [5,6,7]. The changes in the position of the visual model indicate that the familiarity of the route plays a key role in determining the visual sampling strategy [8]. Considering the effects of road familiarity on distracted driving behavior and operation when driving on a familiar road, drivers’ focus on the object is the most common distracted driving behavior. Some research shows that there is significant impairment to driving resulting from the shift of attention from the task of operating the vehicle and that these impairments are directly related to the cognitive workload of these on-board activities [9,10]. When considering the effects of distraction on driver behavior characteristics, a significant difference exists between different distracting tasks and distraction levels. When considering the three conditions of no task, easy cognitive task, and difficult cognitive task, the comparison of “no task” and “difficult task” shows the most obvious change in visual behavior. When looking out of the car, the driver spends more time in the front center and less time looking around [11]. Different types of drivers have different effects when faced with stress and distraction [12]. Cognitive workload tasks are usually performed by N-back, mathematical problem calculation, and plate billboard memory. To address this problem, many studies have been dedicated to this domain through on-road driving tests and simulation experiments. Two different road environments are designed, simple and complex, through comparative simulation experiments to study the impact of interference on the driving performance of the driver and it has been found that the vehicle interference task causes the driver to become distracted, reduces their overall driving performance and reaction to danger, and increases their subjective workload [13].

Many advanced driver assistance systems also detect driver distractions. The AutoRate system can use the front camera of the smartphone installed on the windshield to monitor the driver’s attention [14]. The Berkeley DeepDrive Attention (BDD-A) Dataset contains dashboard video and manual demonstrations with time-stamped sensors and measurement results collected during urban driving in various weather and light conditions. Each participant completed the task for 200 driving videos. From the perspective of machine vision, this predicted the driver’s attention [15,16]. Fernández-Isabel [17] introduced the VISUVER framework, which uses finite state machines to automatically represent and visualize dynamic human behavior. This behavior can be provided by actual or simulated data collected by specific sensors. One of the most effective ways to record the driver’s attention is to evaluate their eye behavior and record the time it takes the driver to look away [18]. The misallocation of driver visual attention has been suggested as a major contributing factor to vehicle accidents. One possible reason is that the relatively high cognitive demands of driving, such as the complex external environment or the distractions in the car, etc., limit the driver’s ability to efficiently allocate gaze [19]. At the same time, driving experience is also a factor that causes changes in visual attention. When performing better on visual attention tasks, this is usually accompanied by eye movements and driving behavior (usually related to safe driving). New drivers cannot scan the road environment like experienced drivers and they will be more dangerous in the event of an emergency. The appearance of other road users on the trajectory that may cause collisions attracts the attention of all drivers, but new drivers are more susceptible to this kind of harm than experienced drivers [20]. Dynamic billboards significantly increase drivers’ scope and attention span, greatly increasing the risk of driving [21]. By simulating drivers’ visual characteristics when overtaking on the highway in a driving simulator, drivers focus on the gaze duration, the saccade duration, the saccade angle following a normal distribution pattern, and the primary visual area [22]. Other studies have conducted a driving simulation test on the five-digit (simple) or 11-digit (complex) visual digital judgment task of drivers and have recorded eye movements with an eye tracker. When the in-vehicle task requires high information processing, drivers’ off-road fixation is frequent [23]. Performing the single and multiple secondary tasks under naturalistic driving, the old people are less likely to finish the tasks than young drivers [24].

At present, driving behavior research mainly adopts various technologies to evaluate drivers’ eye movement information, such as bioelectricity (such as ECG, EMG, and EEG signals data through the use of cameras), head infrared sensors, and eye trackers. Some studies have compared eye movements and dangerous reaction times between simulated driving tasks and similar but video-based passive danger perception tasks. They found that during active driving, participants scanned the road less and fixed their gaze near the front of the vehicle [25]. When sending and receiving emails on a pad in the vehicle, sending emails greatly weakened drivers’ attention and reduced their ability to control the vehicle. Meanwhile, the time spent looking at the display screen greatly increased [26]. The changes of drivers’ gaze are observed by adjusting the radio in the car in different ways on the highway and classifying different drivers through the hidden Markov model. The voice input adjustment method has been found to be safe [27]. According to the eye movement data reported by 100 vehicles, drivers’ gaze changes during the state of fatigue, radio adjustment and conversation are revealed, and such changes within six seconds are predicted through the HSMM model and RNN [28]. The impact of hands-free phones on eye movement patterns when driving is that the eyes gaze less at road signs, other vehicles, and speedometers. The spatial distribution of eye gaze is wide during hands-free calling. When hands-free phones are not used, the line-of-sight distribution depends to a certain extent on changes in driving tasks [29].

**Figure 1:**
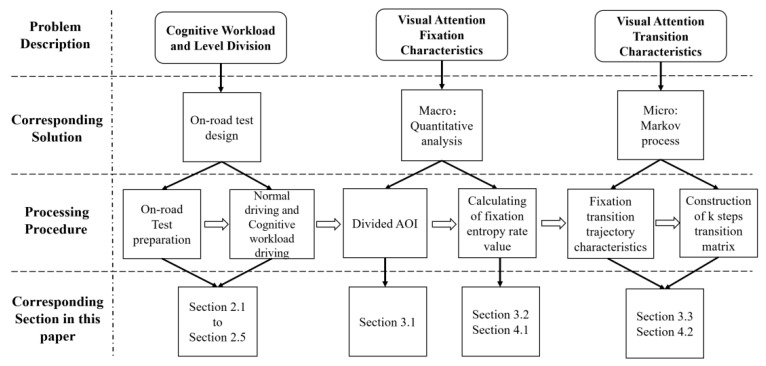
Research framework.

In summary, previous works in this domain have accomplished a series of achievements in the study of driving workload, visual attention distribution, and transition. For visual attention, previous studies have focused on the changes of eye movements on the radio inside the vehicle, billboards outside the vehicle, and in-vehicle gestures, etc. At present, studies have only looked at macroscopic cognitive distraction and there is a lack of in-depth exploration of visual attention under different degrees of cognitive distraction. Moreover, most tests have been conducted in a driving simulation environment and few have performed on-road driving experiments. In-depth studies on the characteristics of drivers’ gaze fixation and transition under cognitive workload conditions are lacking and the relationship between visual and cognitive attention has not been fully explored. In view of these research shortcomings, the participants generated three different levels of cognitive workload through the calculation of math problems of different difficulties. We designed the on-road driving experiment under a real driving environment, exploring the driver’s visual attention fixation characteristics through visual entropy theory and researching the driver’s visual transition characteristics through Markov one-step and two-step transition matrix under different cognitive workloads. The main research ideas are illustrated in Figure 1.

## 2. Experimental Design

### 2.1. Participants

Many studies have proved that subject to the constraints of experimental conditions, small samples can also be used in driver characteristic tests. Usually, the number of samples is greater than 6 to be effective [30,31,32,33]. Fourteen drivers were selected for the on-road driving test and 10 of them completed the experiment, including six male drivers and four female drivers. A questionnaire was used to obtain information and to assess the basic situation of the participants before the test. The age distribution ranged from 25 to 55 years old (Mean = 34; Standard Deviation (SD) = 8.65), with different driving experiences (Mean = 7.0; SD = 6.96) and driving mileage (Mean = 5.72; SD = 6.37). The test required all the drivers to have good driving manners; good vision after correction; good health; no visual and hearing impairment; sufficient rest the day before the test; no intake of stimulating food, such as alcohol and caffeine; and be non-smokers. The demographic information is shown in Table 1.

### 2.2. Apparatus

**Figure 2:**
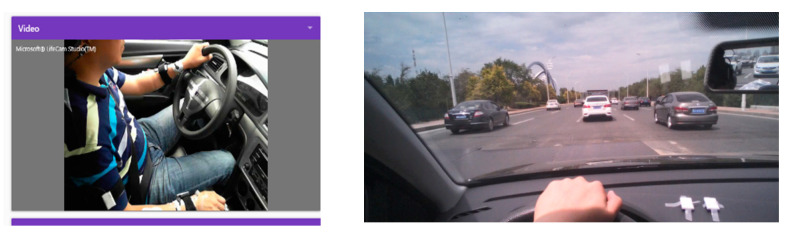
Experiment process record.

The test vehicle was a 2017 Sagitar 1.6T (FAW-Volkswagen, Changchun, China), equipped with an advanced data acquisition system to capture data related to drivers’ action and status in real time. The data acquisition system includes a laser distance sensor (INSIGHT-200A model, with a range of 0.5–200 m, ranging accuracy of ±0.5–1 m, measuring frequency of 10–50 Hz, input voltage of 6–24 V, and a laser wavelength of 905 nm); radar rangefinder (Fluke 419D, with a range of 0–80 m, range accuracy of ±1 mm, and laser wavelength of 635 nm, which can memorize 20 results); Tobii Glasses 2 wearable eye tracker (sampling frequency of 50 Hz/100 Hz, four eye movement cameras, automatic parallel parallax correction, 82° angle of view with a vertical angle of 52°); and BIOPAC MP160 16-channel physiological recorder (a data acquisition & analysis device for ECG, HRV, EEG, EMG, EGG et al. Maximum sampling rate of 400 KHz and accuracy of ±0.003%). The four synchronized camera outputs include the front and rear of the vehicle, drivers’ face and in-vehicle operation video, drivers’ recorded eye movement, vehicle running characteristics, and traffic environment information for the whole process. The drivers operate in accordance with their own daily driving behaviors and habits, thereby collecting their natural driving behavior in a real environment, as shown in Figure 2.

The drivers fix their vision on a certain area during the driving process and transfer such vision between different areas. The visual attention fixation characteristics are analyzed by recording drivers’ gaze duration. The visual attention transfer characteristics are studied by recording the horizontal and vertical coordinates of the fixation target.

### 2.3. Driving Tasks

Participants were required to work in strict accordance with the instructions of the research staff during the test. Under normal driving circumstances, drivers perform the main driving task; that is, to control the vehicle to drive at different speed limits on different sections and carry out the prescribed operations as prompted. In this experiment, the method of listening to and performing mathematical calculations was selected. All the participants had a bachelor’s degree or above and good mathematical calculation ability. According to the difficulty level of the questions, they can be divided into three levels: simple, general, and complex. At the same time, three levels of cognitive workload are defined, namely, mild, moderate, and deep. The duration of each cognitive distracted driving test was approximately 2 min and each participant had to complete the tasks twice. During the mathematical calculation of each level, the experimenter flashed the camera and turned off the light, which is used for the calibration of the post-analysis data and keeps records about performance. The interval between the two groups depended on the drivers’ fatigue degree. See Table 2 for details.

The cognitive workload task was set up to allow drivers to experience different levels of cognitive distraction during the driving process. All drivers were told to improve the calculation accuracy as much as possible before the experiment. It was found that the calculation accuracy rate of the simple tasks exceeded 90% and in general, tasks also exceeded 80%, but as for the complex tasks, this was less than 60% and for the driving safety, some participants even gave up and provided a random answer. So, the purpose of setting math problems of different difficulties is to cause different levels of cognitive distraction. This research considers the characteristics of eye movement behavior and the influence of the calculation accuracy is not considered in the actual process analysis.

### 2.4. Experimental Routes

**Figure 3:**
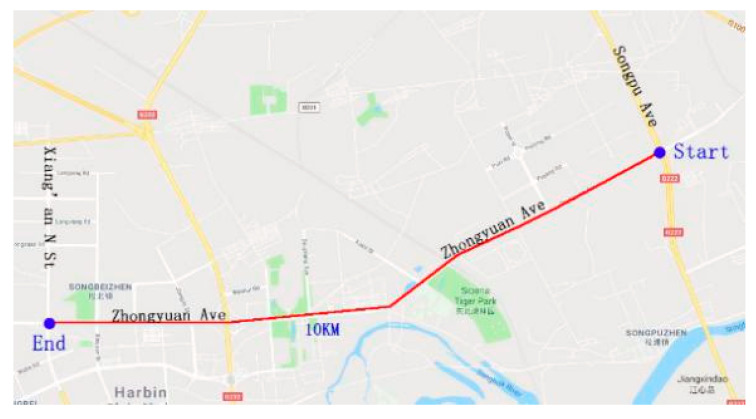
Experimental routes.

A road test was conducted during non-peak hours on weekdays (usually 9:30–11:00 in the morning and 2:30–4:00 in the afternoon). This paper selects the experiment with sunny weather conditions to analyze and when the temperature was suitable for driving. All the test drivers performed the actual vehicle test on the same road section to ensure that all the drivers had similar traffic conditions. Weather and other factors remained mostly consistent during the test. The test road section was selected to be at Zhongyuan Avenue in Harbin. The starting point was the intersection of Songpu Bridge and Zhongyuan Avenue and the ending point was the intersection of Zhongyuan Avenue and Xiang’an North Street. The whole journey was 10 km long and the route is depicted in Figure 3. The test road had six lanes in both directions, separated by the central separation belt from the traffic in the opposite direction. The traffic flow in this section was stable, the road alignment condition was good, the drivers’ field of view was clear, and the cognitive workload test was reasonably safe. No collision accident occurred in all the experiments.

### 2.5. Experimental Process

(1) The experimental staff prepared the test notification. The test subject was informed of the whole process and the subject filled in the personal information form. Then, the staff placed the equipment on the test subject.
(2) The staff guided the drivers into the test vehicle, switched on all the test equipment, and completed the calibration and synchronization of the equipment. Once the drivers were ready, they drove around the safety area for approximately 15 min to become accustomed to the setting.
(3) The participants entered the designated test section to start driving and the staff recorded data from the start time of the test. The participants performed the prescribed operations under the guidance of the staff unless safety hazards arose. Data continued to be collected during the test to capture the driving environment and drivers’ behavior under different environments.
(4) On a specific road section, the drivers performed the cognitive workload task while maintaining the main driving task. They completed the corresponding mathematical calculation according to the experimental design setting. In case hazardous situations occurred, the drivers had the option to terminate the task.
(5) After the test, the staff turned off the test equipment and assisted the test subject to take off the test equipment. The participants completed the questionnaire and received the test compensation (200 RMB). The staff compiled, copied, and archived the test data.

## 3. Methodology

### 3.1. Area of Interest (AOI) Division of Fixation 

**Figure 4:**
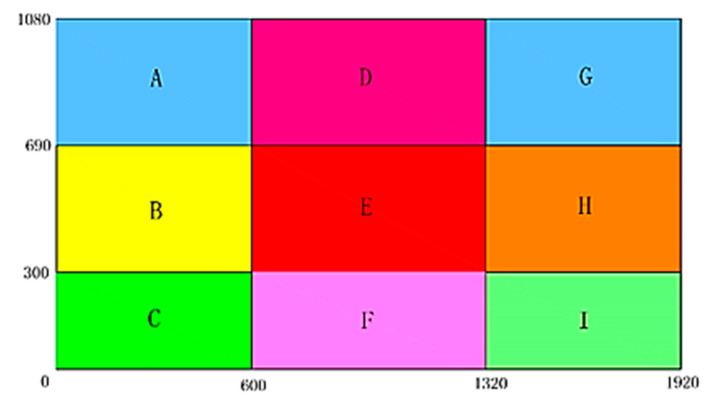
Area of Interest (AOI) division.

Peter et al. [34] divided the line of sight into nine equal non-overlapping fixation areas. Falkmer et al. [35] divided the view area into three categories: distant, front, and near body areas. To cover the special fixation points (left and right mirrors, steering wheel, etc.), the drivers’ fixation interest area was divided into nine categories, as displayed in Figure 4. According to Tobii’s default settings, two or more AOI glances with a blink interval of less than 75 ms were merged from the analysis. Considering that head movement will affect the result, we manually encoded the fixation based on the fixation targets in Table 3, where a glance to the right side is coded as AOI-H, a glance to the left side is coded as AOI-B regardless of the head direction, the rest may be deduced by analogy. This research adopts the identification by Velocity Threshold (IVT), which is an algorithm that can detect fixation easily that functions in this way [36,37]. The fixation targets of each AOI are described in Table 3.

### 3.2. Research Method of Driver Visual Attention Fixation Characteristics

The information entropy method was used to study the visual attention measure of different regions and the randomness of the drivers’ glance is reflected by the magnitude of the entropy rate, which was designed to bridge the gap between the qualitative methods currently used for evaluating vision in driving research and quantitative methods, which are actually desired [38]. The discrete variable entropy information is E, as shown in Equation (1). The maximum value in Equation (2) and the fixation entropy value E*_n_* are given in Equation (3).

(1) $$E=-\sum P_{x_{i}}\log_{2}P_{x_{i}}$$

(2) $$E_{\max}=\log_{2}D$$

(3) $$E_{n}=\sum_{i=1}^{D}\frac{E/E_{\max}}{DT_{x_{i}}}=\sum_{i=1}^{D}\frac{-\sum P_{x_{i}}\log_{2}P_{x_{i}}/\log_{2}D}{DT_{x_{i}}}.$$

D is the number of fixation areas, D = 9; $P_{x_{i}}$ is the fixation probability for a certain area; $T_{x_{i}}$ is the average fixation time of the drivers in a certain area; *i* is the serial number of the region (serial numbers range from 1 to 9).

### 3.3. Research Method of Driver Visual Attention Transition Characteristics

The fixation process is continuous and random and the fixation point continuously moves periodically to form their eye movement. To study the characteristics of fixation transition, considering the correlation among the current, previous, and next fixed gaze behaviors is usually necessary.

Assume that {X(t),t∈T} is a random process. For any positive integer n and continuous time t*_i_*, $P(X(t_{1})=x_{1},\ldots ,X(t_{n-1})=x_{n-1})>0$. If the conditional distribution satisfies Equation (4), then X(t) is called a Markov process.

(4) $$P\{X(t_{n})\leq x_{n}|X(t_{1})=x_{1},\ldots ,X(t_{n-1})=x_{n-1}\}=P\{X(t_{n})\leq x_{n}|X(t_{n-}{}_{1})=x_{n-1}\}$$

The drivers’ visual transition characteristics are studied by constructing a Markov transition matrix. When the steady state is reached, the steady distribution of fixation can reflect the probability of each area during driving. By solving the steady distribution of the matrix, the degree of drivers’ attention to each area can be determined when the time is long enough.

One-step transition is defined as the transition of a fixation point from the previous to the current fixation point or from the current fixation point to the next. The probability of this transition is called one-step transition probability. The process in which the fixation point is transferred from the previous fixation point to the next through the current fixation point is defined as a two-step transition. Its probability is called two-step transition probability. The K step transition probability matrix is represented by P(k).

(5) $$P(k)=\left[\begin{array}{cccc} p_{11} & p_{12} & \cdots & p_{1n}\\ p_{21} & p_{22} & \cdots & p_{2n}\\ \vdots & \vdots & \vdots & \vdots \\ p_{n1} & p_{n2} & \cdots & p_{nn} \end{array}\right].$$

$p_{ij}$ is the element of the i-th row and the j-th column of the transition probability matrix. The sum of the transition probabilities of each row is 1.

The Markov chain has ergodicity. After a period, the system can reach a stable state. After a certain transition duration in the Markov process, the probability of reaching *j* tends to be stable from the initial state *i*. If a state probability vector $X=(x_{1},x_{2},\ldots ,x_{n})$ exists, then make XP(k)=X; where X is called the stationary distribution of P(k). That is,

(6) $${\left[\begin{array}{cccc} p_{11} & p_{12} & \cdots & p_{1n}\\ p_{21} & p_{22} & \cdots & p_{2n}\\ \vdots & \vdots & \vdots & \vdots \\ p_{n1} & p_{n2} & \cdots & p_{nn} \end{array}\right]}^{T}(\begin{array}{c} x_{1}\\ x_{2}\\ \vdots \\ x_{n} \end{array})=(\begin{array}{c} x_{1}\\ x_{2}\\ \vdots \\ x_{n} \end{array})$$

(7) $$x_{1}+x_{2}+\cdots +x_{n}=1$$

## 4. Results and Discussion

### 4.1. Analysis of Drivers’ Visual Attention Fixation Characteristics

Visual entropy is the subjective measure of the human eye on image information, which can reflect the driver’s visual attention. When E = 0 and $P_{x_{i}}=1$, entropy rate value En takes the minimum value of 0, indicating that during the driving process, drivers’ fixation is concentrated on one area while ignoring other areas. If the focus is always on the central area, then visual attention is only concentrated on the road ahead. The overall traffic environment is weak and the safety hazard is small under the condition of simple traffic conditions. If the focus is on the road ahead for a long time, then identifying unexpected situations is difficult without observing other areas and drivers may properly look away from the forward road scene to decrease the likelihood of safety hazards due to unexpected hazards. If the vision is focused on other areas, then dangerous situations and traffic accidents may also occur.

**Figure 5:**
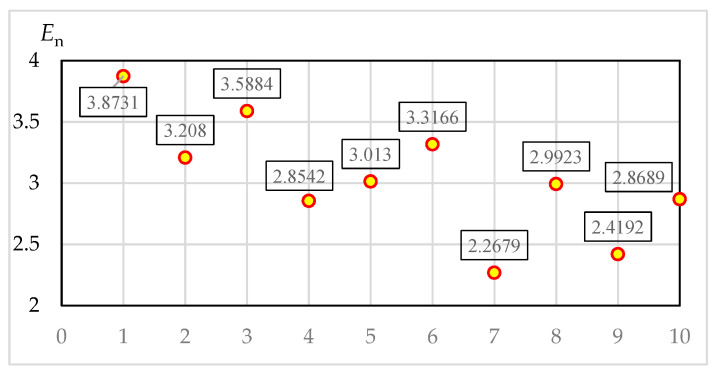
Fixation entropy value of each driver.

In this study, the visual entropy of the entire driving process is statistically analyzed from a macro level. After data processing and analysis, the fixation duration and probability of each region of the 10 test subjects are shown in Table 4 (abnormal data are much lower than other values, which are marked in gray), as presented in Table 5. The entropy value of each driver’s fixation is illustrated in Figure 5.

**Figure 6:**
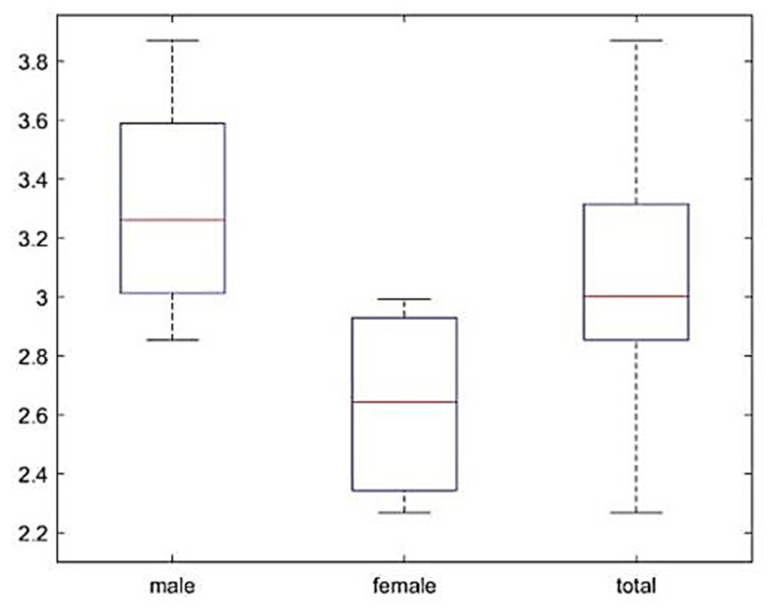
Comparison of various types of drivers.

The average fixation duration can reflect drivers’ attention in each region at the time. Table 4 shows that most drivers can reasonably allocate the fixation duration of each area. Through the anomaly data, a small number of drivers have a short staying time on the left-view, far-left, and far-right mirrors. Therefore, drivers do not give enough attention to targets further away from them. Table 5 presents that drivers have the largest fixation probability on the area near the front. The gaze probability on the overall area in front is more than 90%, indicating that each driver has a clear gaze in front most of the time. No. 1–6 are male drivers, whereas No. 7–10 are female drivers. As displayed in Figure 6, the average entropy rate of male drivers exceeds 3.2, whereas the average value of female drivers is just above 2.6, with a difference of 23.08%.

**Figure 7:**
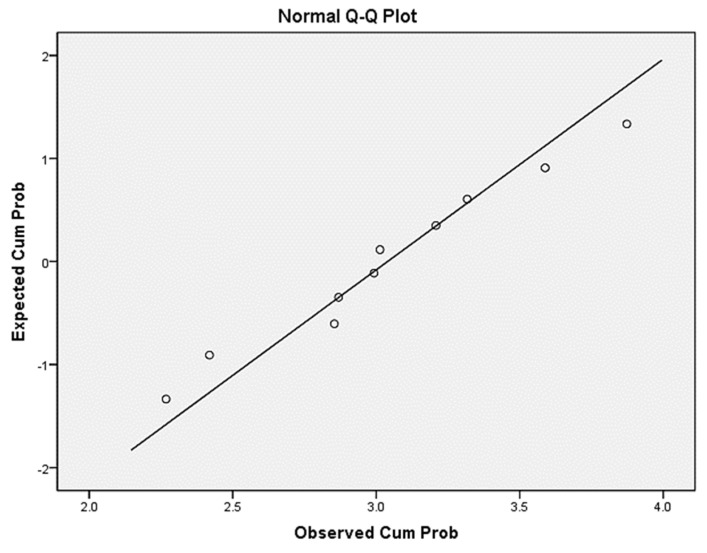
Normality test Q-Q diagram.

The samples tested for normality are shown in Table 6 and Figure 7. The average En value of the sample is 3.04016, the median is 3.0027, and the sample size is 10. Based on the Kolmogorov-Smirnov test results, sig. = 0.2 > 0.05, which is subject to normal distribution. The Q-Q test in Figure 7 reveals that all points are located near the straight line and the sample is normal. The fixation entropy rate of driver No. 5 is closest to the mean and median values. The sample has normality; thus, the results of the visual transition characteristics analysis of driver No. 5 are selected to reflect the regularity of the overall sample.

### 4.2. Analysis of Drivers’ Visual Attention Transition Characteristics under Different States

#### 4.2.1. Visual Transition Trajectory Characteristics

**Figure 8:**
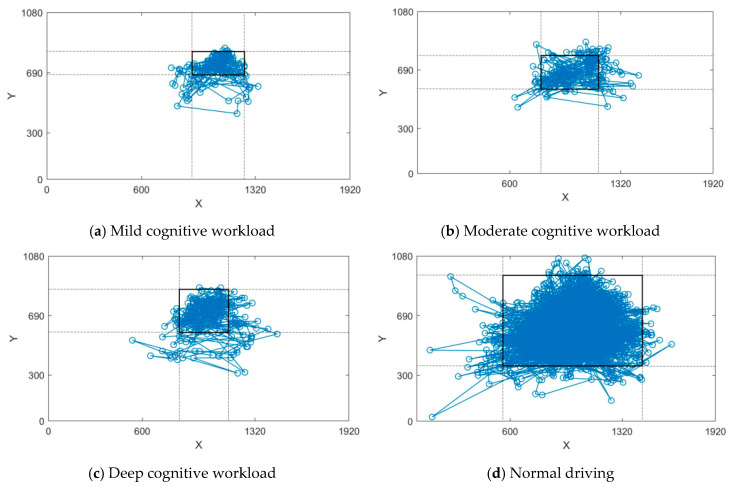
Driver’s fixation transition trajectory under different states. (**a**) Mild cognitive workload (**b**) Moderate cognitive workload (**c**) Deep cognitive workload (**d**) Normal driving. Note: In order to better display the data, we use squares to indicate areas where the data are concentrated.

The fixation trajectory of drivers in mild, moderate, and deep cognitive workloads along with normal driving state is displayed in Figure 8. The horizontal and vertical coordinates indicate the range of the fixation points recorded by the eye-movement apparatus. From the area of eye diversion, the drivers’ vision is mainly concentrated on the front during the whole test. In normal driving, drivers have the widest coverage of the current driving lane, covering almost the front and far areas, however maybe they were just bored in the normal driving task and looked around more. When mild cognitive workload occurs, fixation transition mainly focuses on the distant area directly in front. As the workload level increases, the fixation transition area gradually shifts to the junction between the near and distant areas located directly in front.

**Figure 9:**
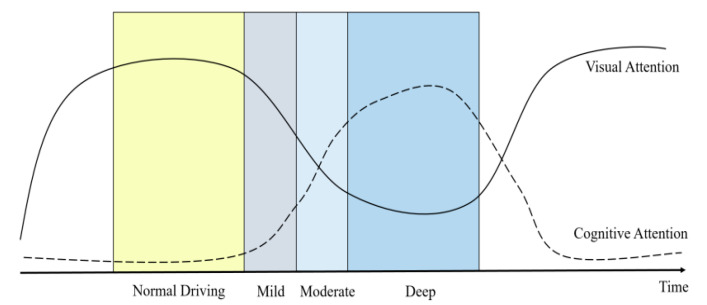
Schematic diagram of the competition between visual and cognitive attention under different states.

Based on previous works [39] and the outcome of this study, visual and cognitive rules occupying drivers’ attention in different states are obtained. As illustrated in Figure 9, the cognitive and visual effects on drivers’ total attention can be derived; in normal driving conditions, drivers can pay close attention to all areas due to sufficient visual resources. When mild cognitive workload occurs and occupies attention resources, the workload is lower than the visual resources. Drivers can also look ahead and ensure safe driving. As the degree of cognitive workload increases, cognitive resources gradually exceed visual resources. To drive safely, drivers must focus on the front and the far junction area and continue to have a wide range of line-of-sight movements. The degree of workload is deepened and the hidden dangers of driving safety increase.

#### 4.2.2. Visual Attention Transition Characteristics in Various Areas

**Figure 10:**
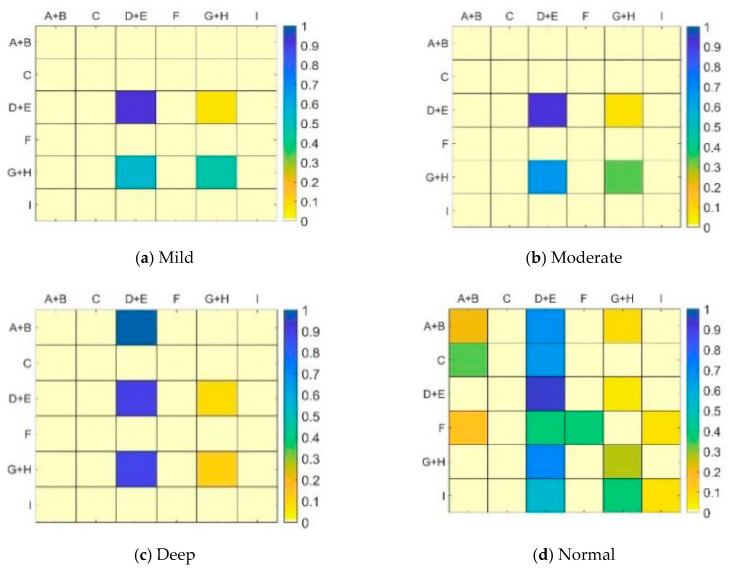
One-step transition probability matrix under different states. (**a**) Mild (**b**) Moderate(**c**) Deep (**d**) Normal.

**Figure 11:**
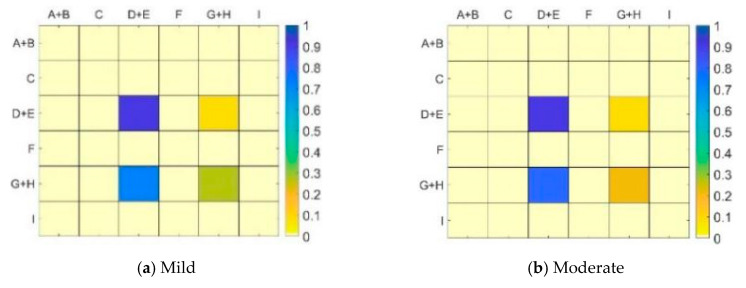

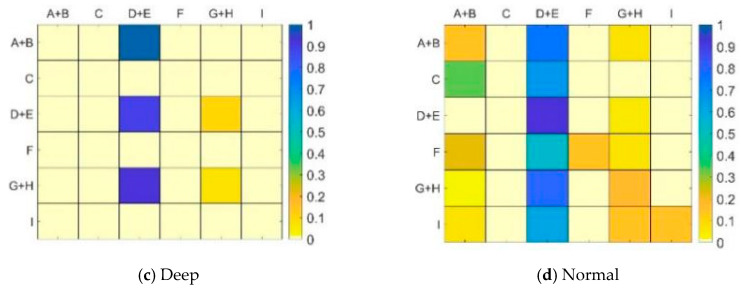
Two-step transition probability matrix under different states. (**a**) Mild (**b**) Moderate(**c**) Deep (**d**) Normal.

Areas A and B belong to the left side of the current driving lane; areas D and E belong to the front of the current driving lane; and areas G and H belong to the right side of the current driving lane. These areas are divided for analysis in the statistical data. The one- and two-step transition probability matrices of drivers’ vision in each fixation area are obtained by the statistics of drivers’ vision in mild, moderate, and deep cognitive workloads and normal driving states. Figure 10a–d and Figure 11a–d present these details.

The one-step transition probability matrix can reflect the position change of drivers’ vision at a continuous moment, whereas the two-step transition probability matrix can reflect the looking back of drivers. Whether the line of sight returns to the same area after two shifts is observed; that is, whether a certain area needs a continuous gaze.

By comparing Figure 10 and Figure 11, certain similar points can be noted. For example, drivers’ vision has the highest probability of shifting from the front to the front (Area D+E to D+E) in different states, and such a shift is above 0.9. In the normal driving state, drivers can visually search and observe the traffic environment well. When performing cognitive workload tasks, drivers show obvious rigidity in their visual search and the fixation point is mainly shifted from the fixation area in front to the right. As the degree of cognitive workload increases, the number of times the drivers’ vision stays in the fixation area in front of the current lane decreases. When the cognitive workload task is performed, the main task of drivers is lane maintenance and safe driving with the vehicle. The reduction of attention to the front fixation area undoubtedly increases the risk of driving.

Further comparison of Figure 10 and Figure 11 can reveal certain differences. Table 7 and Table 8 show the areas and differences in the one- and two-step transition probabilities of the drivers’ vision under different states, respectively. During normal driving, the probability of the drivers’ vision shifts from the in-vehicle to the left side and the front increases by 49.97% and 40.02%, respectively. The proportion of moving inside the vehicle decreases by 60.01%. The large probability of one-step transition indicates that the information in the vehicle is complex and staying at multiple consecutive gaze points is necessary to obtain information. The probability of two-step transition drastically dropped, indicating that drivers’ fixation on the interior of the vehicle is not a continuous behavior. Even if a short stay exists, the vision can be transferred to the left and front areas in time. The proportion of attention to the right-view mirror also doubles, indicating that continuous attention is required when observing the right-view mirror. In mild and moderate workloads, the proportion of drivers’ vision transition from the right side to the front area increases. By contrast, the proportion of continuous staying in the right area is significantly reduced, indicating that under the influence of mild and moderate cognitive workloads, drivers’ eyes still shift to the right front area, but the proportion of improvement decreases as workload increases. In the case of depth workload, no significant change is observed in the one- and two-step transition probabilities of the drivers’ vision.

#### 4.2.3. Visual Attention Characteristics in Steady State

Assuming that the drivers’ visual attention transfers to a sufficient number of times, a steady state is observed in which their visual attention can be analyzed. The steady distribution of fixation is shown in Equation (8). Note that X1, X2, X3, and X4 represent the steady distribution values under mild, moderate, deep cognitive workloads, and normal driving state.

(8) $$X_{1}=\left[\begin{array}{c} 0\\ 0\\ 0.8947\\ 0\\ 0.1053\\ 0 \end{array}\right]X_{2}=\left[\begin{array}{c} 0\\ 0\\ 0.8966\\ 0\\ 0.1034\\ 0 \end{array}\right]X_{3}=\left[\begin{array}{c} 0.0035\\ 0\\ 0.9031\\ 0\\ 0.0934\\ 0 \end{array}\right]X_{4}=\left[\begin{array}{c} 0.011\\ 0.0004\\ 0.9193\\ 0.0019\\ 0.0655\\ 0.0019 \end{array}\right].$$

From the perspective of the smooth distribution value in the normal driving state, the fixation point of drivers is scattered to each fixation area, with the largest probability of occurrence in the front of the current lane, followed by the right of the current lane. In mild and moderate cognitive workloads, drivers’ fixation point can only appear in the front and right lanes of the current lane. In deep cognitive workload, drivers’ gaze can also have a small probability to focus on the left lane.

**Figure 12:**
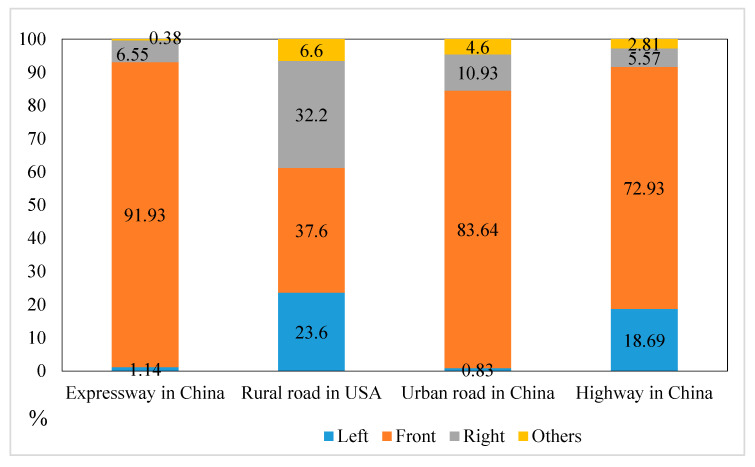
Comparison of different literature fixation areas.

FALKMER research shows that both urban roads and rural roads increase the driver’s cognitive needs, but after the driver enters the city, they pay more attention to the outside world and require a higher level of cognitive attention [40]. Figure 12 illustrates a comparison of various works on rural roads [41], the Chinese urban trunk roads in [42], and the Chinese highways in [43]. Due to different AOI areas in different literature, AOI areas are divided according to the left, front, right, and other areas of fixation. Reference [42] and [43] conducted the real vehicle test, whereas Bao [41] performed the driving simulation test. Considering the situation in China and the United States, the attention of Chinese drivers to the front is more than twice that of American drivers by percentage points. When driving on urban roads, the attention to the front is higher than that on the highway. In the real road driving environment, the visual attention of the driver bears more mental workload than the simulated environment [44]. A significant difference also exists between the results from the driving simulation and actual driving environment, and some researchers have also confirmed this view [18,21,45]. In actual driving, the driver bears a greater risk than the driving simulator, with drivers focusing on the forward transition in the real driving environment.

## 5. Limitations and Future Work

The study has certain limitations and the following problems will be solved in future research:

(1) At present, we only studied the overall accuracy of mathematical calculations under different cognitive workload conditions, but did not consider the relationship between mathematical calculation accuracy and driver’s visual attention. Future research will take calculation accuracy as an indicator to analyze the influence of different cognitive workloads on such accuracy.
(2) At present, there is no analysis of the visual entropy of different cognitive levels, but only the whole process of driving. The next step will be to explore its changes under different levels of cognition.
(3) Other comprehensive eye movement indicators, such as pupil diameter change and blink frequency, can be considered to analyze the visual attention of drivers under the cognitive workload condition. Dynamic AOI division will also be adopted for research.
(4) More participants will be recruited to explore the visual attention characteristics of drivers under cognitive workload and we will add some driving simulation experiments for supplementary comparative analysis.
(5) At present, we have generated three different degrees of cognitive workload for participants through the calculation of mathematical problems. In future work, we will quantify the indicators and give specific definitions for the three different levels.

## 6. Conclusions

Our findings are summarized into the following observations:

(1) Drivers’ fixation entropy rate values for each area are calculated from the experimental data and their visual attention is measured by a quantitative method. The comparison between male and female drivers shows that the mean fixation entropy rate of males is 23.08% higher than that of female drivers.
(2) Under normal circumstances of driving, drivers can cover almost the front fixation area. In the case of mild workload, drivers’ eyes are mainly focused on the distant area directly in front. As cognitive workload increases, the area where the eyes are shifted moves toward the junction between the near and far areas directly in front. The relationship between cognitive and visual occupying attention resources is also analyzed. When driving normally, visual resources are sufficient. When cognitive workload occurs, visual and cognitive resources have a competitive relationship. As the degree of cognitive workload increases, cognitive attention occupies resources more than the visual.
(3) Under normal circumstances of driving, drivers’ vision can be transferred well among various areas. Under mild and moderate cognitive workloads, drivers’ eyes shift between the front and right of the current lane and the number of times the drivers’ eyes stay in the fixation area in front of the current lane decreases as workload increases.
(4) Under normal circumstances of driving, in-vehicle transfer is not a continuous behavior. Eyes shift to the left and the front area very quickly. In mild and moderate workloads, the proportion of drivers’ vision transition from the right side to the front area is increased, whereas the proportion of continuous stay in the right area is significantly reduced. Therefore, under the influence of mild and moderate cognitive workloads, drivers’ eyes still shift to the right and front areas. However, the proportion of improvement decreases as workload increases. In the case of in-depth workload, no significant change is observed in the one- and two-step transition probabilities of the drivers’ vision.
(5) The geometry of the road is not considered because the driver’s line of sight is on the curve or tangent of the road and the effect on dynamic vision exists. This issue will be further considered in the next work.

## Figures and Tables

**Table 1:** Basic information description of the participants.

Driver Characteristics	All of the Participants			
	Mean	Standard Deviation	Number (Person)	Percentage (%)
**Gender**	—	—	10	—
Male	—	—	6	60
Female	—	—	4	40
**Age (Year)**	34.00	8.65	10	—
≤25	25.00	0.00	2	20
(25, 40)	32.30	4.61	6	60
≥40	48.00	5.00	2	20
**Driving Experience (Year)**	7.00	6.96	10	—
≤2	1.67	0.47	3	30
(2, 8)	3.67	0.94	3	30
≥8	13.50	6.95	4	40
**Driving Mileage (10,000 Km)**	5.72	6.37	10	—
≤2	0.93	0.58	4	40
(2, 5)	3.27	1.03	3	30
≥5	14.57	4.35	3	30
**Collision Accidents in the Past Three Years**	—	—	10	—
None	—	—	6	60
Once	—	—	3	30
More than Once	—	—	1	10
**Educational Background**	—	—	10	—
Bachelor	—	—	2	20
Master	—	—	6	60
PhD	—	—	2	20
**Using Mobile Phone During Driving**	—	—	10	—
Hardly Ever	—	—	3	30
Sometimes	—	—	5	50
Always	—	—	2	20

— indicates an indicator that cannot be quantified.

**Table 2:** Description of cognitive workload task under different levels.

Tasks	Level	Type and Example	Question Number	Total Number	Limited Time for Each Question (s)
Simple	Mild	Addition calculation of 2-digit positive numbers without carry (21 + 23 =)	3	5	6
		Subtraction calculation of 2-digit positive numbers without borrowing (25 − 12 =)	2		
General	Moderate	Addition calculation of 2-digit positive numbers with carrying (16 + 17 =)	2	3	10
		Subtraction calculation of 2-digit positive numbers with borrowing (54 −18 =)	1		
Complex	Deep	Subtraction calculation of 2-digit negative numbers (18 − 33 =)	1	2	15
		Continuous addition calculation of 2-digit positive numbers (12 + 23 + 36 =)	1		

**Table 3:** Description of AOI area and fixation target division.

Serial	Fixation Area		Fixation Target
A	Left side	Distant area on the left side	Vehicles and traffic signs far from the left side or opposite lanes
B		Near area on the left side	Vehicles and traffic signs near the left side or opposite lanes
C		Bottom left side	Left mirror and nearby area
D	Front side	Far ahead	Distant area of the current driving lane
E		Near area in the front side	Near area of the current driving lane
F		In-vehicle area	In-vehicle targets, including on-board devices such as dashboard steering wheel
G	Right side	Distant area on the right side	Vehicles and traffic signs far away from the right side, rearview mirrors
H		Near area on the right side	Vehicles and traffic signs near the right side
I		Bottom right side	Glove box, right mirror, and nearby area

**Table 4:** Statistics of driver fixation duration.

Area	Driver Number									
	1	2	3	4	5	6	7	8	9	10
	Average Fixation Duration (ms)									
A	126.46	125.26	136.20	125.75	205.37	90.10	133.60	0.04	163.33	100.91
B	135.82	149.60	162.55	103.13	231.01	135.96	140.03	113.68	183.41	188.57
C	150.91	80.00	111.11	0.09	100.10	170.00	0.12	220.04	103.33	220.00
D	153.32	231.81	184.57	194.81	239.28	125.58	116.56	92.51	161.86	98.18
E	175.58	251.43	218.14	185.45	217.37	141.99	145.83	163.95	184.77	176.83
F	195.96	173.76	192.40	146.51	259.27	158.31	108.81	147.43	143.76	133.75
G	163.94	80.00	108.24	148.72	102.18	125.93	97.50	60.00	111.11	0.09
H	156.79	113.96	131.83	129.12	101.05	123.11	147.85	168.13	173.83	190.18
I	170.59	138.57	137.53	171.43	120.01	137.04	113.33	121.25	120.87	116.67

**Table 5:** Statistics of the fixation probability of each region.

Area	Driver Number									
	1	2	3	4	5	6	7	8	9	10
	Regional Fixation Probability (%)									
A	2.21	1.21	1.24	1.49	17.18	0.12	1.23	0.01	0.19	0.12
B	3.76	0.49	1.39	0.45	2.86	3.39	0.10	0.78	0.74	1.06
C	0.68	0.01	0.09	0.01	0.02	0.08	0.01	0.12	0.06	0.09
D	38.66	59.72	48.87	13.13	8.91	9.16	2.75	0.13	1.37	0.44
E	44.61	35.05	39.67	76.51	68.41	75.08	86.91	76.25	74.13	61.91
F	2.93	2.11	2.62	0.86	1.91	6.01	2.36	3.93	1.95	1.73
G	2.98	0.36	0.33	5.06	0.50	0.78	0.29	0.01	0.10	0.01
H	3.37	0.78	1.44	1.00	0.17	4.53	5.83	18.04	21.16	34.34
I	0.80	0.25	4.34	1.47	0.02	0.85	0.50	0.70	0.27	0.28

**Table 6:** Normal distribution test result of fixation entropy rate value.

**Type**	**The Statistics of Basic Description**						
**Items**	***N***	**Mean**	**Median**	**SD**	**SEM**	**Kurtosis**	**Skewness**
*E*_n_	10	3.04016	3.03679	0.4885195	0.1544834	−0.182	0.068
**Type**	**The Results of Normal Distribution**						
**Item**	**Kolmogorov-smirnov**				**Shapiro-wilk**		
	Statistical data		df	Sig.	Statistical data	df	Sig.
*E*_n_	0.152		10	0.200	0.975	10	0.933

**Table 7:** Description with significantly increased under different states.

State	Mild	Moderate	Normal Driving			
Transition Area	G + H to D + E	G + H to D + E	F to A + B	A + B to D + E	F to D + E	I to I
One-step	0.5455	0.6667	0.1539	0.6753	0.3846	0.0769
Two-step	0.7273	0.7826	0.2308	0.7532	0.5385	0.1538
Increase Proportion	33.31%	17.38%	49.97%	11.54%	40.02%	100%

**Table 8:** Description with significantly reduced under different states.

State	Mild	Moderate	Normal Driving			
Transition Area	G + H to G + H	G + H to G + H	A + B to A + B	F to F	G + H to G + H	I to G + H
One-step	0.4545	0.3333	0.2078	0.3846	0.2680	0.3846
Two-step	0.2727	0.2174	0.1558	0.1538	0.1808	0.1538
Reduce proportion	40%	34.77%	25.02%	60.01%	32.54%	60.01%
